# Investigation of the blood glucose target for mortality reduction by means of bedside-type artificial pancreas

**DOI:** 10.1186/cc9816

**Published:** 2011-03-11

**Authors:** M Hoshino, Y Haraguchi, I Mizushima, M Sakai, K Oda, S Kajiwara, M Takagi

**Affiliations:** 1 Shisei Hospital, Saitama, Japan; 2 National Hospital Organization Disaster Medical Center, Tokyo, Japan; 3 Nippon Engineering College, Tokyo, Japan; 4 Tokyo Women's Medical University Hospital, Tokyo, Japan

## Introduction

The blood glucose (BG) target has not been determined especially in acutely ill patients. The purpose was to investigate the BG target in order to reduce the mortality in terms of clinical phases (Early (E) phase and Late (L) phase) as well as to clarify mutual relationships among the BG parameters.

## Methods

Patients with daily mean BG (BGm) below 200 mg/dl in whom BG was controlled by a bedside-type artificial pancreas (AP), STG22, were researched in the E phase (3.3 ± 2.5 days after admission, *n *= 67) and L phase (10.1 ± 3.4 days after admission, *n *= 77). Nutritional support for all the patients was performed by total parenteral nutrition. Studied items: BG parameters (mg/dl; BGm, daily standard deviation of BG (BGsd), daily maximal and minimal BG (BGmax, BGmin), and daily BG difference (BGd: BGmax - BGmin)), maximal value of the accuracy (%) of the BG parameters for predicting survival (AS), and correlation coefficients (*r*) among the BG parameters.

## Results

AS (%): E phase/L phase; BGm below 196 (75%)/BGm below 175 (68%), BGsd below 17 (73%)/BGsd below 20 (62%), BGmax below 225 (72%)/BGmax below 218 (65%), BGmin below 172 (72%)/BGmin below 158 (73%), and BGd below 80 (70%)/BGd below 98 (68%). Strong positive correlation (*r*) was found in both phases (E phase/L phase) between BGsd and BGd (*r *= 0.87/*r *= 0.95), BGsd and BGmax (*r *= 0.79/*r *= 0.78), and BGd and BGmax (*r *= 0.77/*r *= 0.82). There was no significant correlation in both phases (E phase/L phase) between BGm and BGsd (*r *= 0.16/*r *= 0.37), BGm and BGd (*r *= 0.13/*r *= 0.38), and BGmax and BGmin (*r *= 0.07/*r *= 0.29).

## Conclusions

The above-mentioned values of the BG parameters were considered to be the BG targets. Strict BG control in the E phase is significant, from the data indicating that the AS values in the E phase were greater than those in the L phase except BGmin. BGm, BG variability (BGsd, BGd), and BGmin were suggested to be independent BG parameters. AP was essential for determining the BG target.

